# Bis[5-chloro-2-(prop-2-yn-1-yl­oxy)phen­yl]methane

**DOI:** 10.1107/S1600536811003205

**Published:** 2011-01-29

**Authors:** Qamar Ali, Itrat Anis, M. Raza Shah, Seik Weng Ng

**Affiliations:** a H.E.J. Research Institute of Chemistry, International Center for Chemical and Biological Sciences, University of Karachi, Karachi 7527, Pakistan; b Department of Chemistry, University of Malaya, 50603 Kuala Lumpur, Malaysia

## Abstract

The mol­ecule of the title compound, C_19_H_14_Cl_2_O_2_, has two benzene rings connected to a methyl­ene C atom, and the rings are aligned at 66.3 (1)°. Inter­molecular C—H⋯π and π–π stacking inter­actions are observed in the crystal structure, the centroid–centroid distances between parallel benzene rings being 3.7529 (12) and 3.6201 (12) Å, respectively.

## Related literature

For a related structure, see: Hussain *et al.* (2009 ▶).
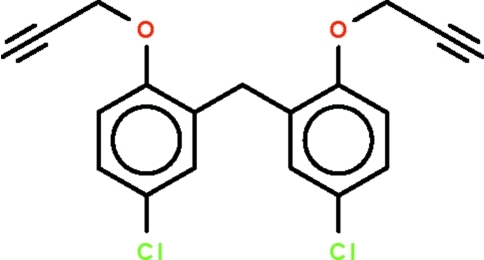


## Experimental

### 

#### Crystal data

C_19_H_14_Cl_2_O_2_
*M* *_r_* = 345.20
Triclinic, 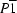

*a* = 8.4844 (5) Å
*b* = 9.7845 (6) Å
*c* = 11.2568 (6) Å
α = 86.258 (5)°
β = 71.412 (5)°
γ = 64.707 (6)°
*V* = 798.08 (8) Å^3^
*Z* = 2
Mo *K*α radiation
μ = 0.41 mm^−1^
*T* = 100 K
0.30 × 0.25 × 0.20 mm

#### Data collection

Agilent SuperNova Dual diffractometer with an Atlas detector
Absorption correction: multi-scan (*CrysAlis PRO*; Agilent, 2010 ▶) *T*_min_ = 0.815, *T*_max_ = 1.000
6118 measured reflections
3523 independent reflections
2975 reflections with *I* > 2σ(*I*)
*R*_int_ = 0.029

#### Refinement

*R*[*F* ^2^ > 2σ(*F* ^2^)] = 0.038
*wR*(*F* ^2^) = 0.099
*S* = 1.00
3523 reflections
216 parameters
2 restraints
H atoms treated by a mixture of independent and constrained refinement
Δρ_max_ = 0.30 e Å^−3^
Δρ_min_ = −0.35 e Å^−3^

### 

Data collection: *CrysAlis PRO* (Agilent, 2010 ▶); cell refinement: *CrysAlis PRO*; data reduction: *CrysAlis PRO*; program(s) used to solve structure: *SHELXS97* (Sheldrick, 2008 ▶); program(s) used to refine structure: *SHELXL97* (Sheldrick, 2008 ▶); molecular graphics: *X-SEED* (Barbour, 2001 ▶); software used to prepare material for publication: *publCIF* (Westrip, 2010 ▶).

## Supplementary Material

Crystal structure: contains datablocks global, I. DOI: 10.1107/S1600536811003205/xu5147sup1.cif

Structure factors: contains datablocks I. DOI: 10.1107/S1600536811003205/xu5147Isup2.hkl

Additional supplementary materials:  crystallographic information; 3D view; checkCIF report

## Figures and Tables

**Table 1:** Hydrogen-bond geometry (Å, °) *Cg* is the centroid of the C11–C16 benzene ring.

*D*—H⋯*A*	*D*—H	H⋯*A*	*D*⋯*A*	*D*—H⋯*A*
C6—H6⋯*Cg*^i^	0.95	2.60	3.471 (2)	153

Symmetry code: (i) 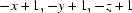.

**Fig. 1.**
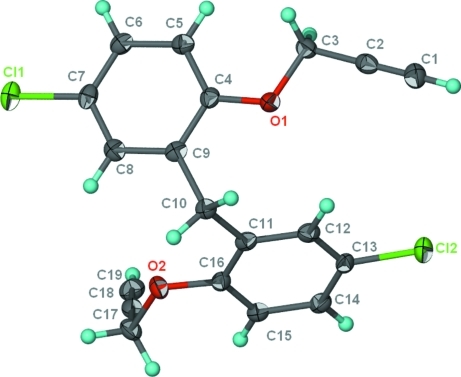
Thermal ellipsoid plot (Barbour, 2001) of C19H14Cl2O2 at the 70% probability level; hydrogen atoms are drawn as spheres of arbitrary radius.

## supplementary crystallographic information

### Comment

We have reported several compounds that adopt a V-shape; this shape is induced by a methylene linkage to two aromatic systems. An example is bis[2-(3-bromopropoxy)-5-methylphenyl]methane, which methyl carbon has a widened angle of 115.0 (2)° (Hussain *et al.*, 2009). The methylene angle in the present compound (Scheme I, Fig. 1) is similar [114.4 (1) °]. Two two aromatic rings that are connected the methylene carbon are aligned at 66.3 (1)°. Intermolecular C—H···π interaction occurs between inversion center related molecules (Table 1). π-π stacking is also present between parallel benzene rings in the crystal structure, centroid-to-centroid distances being 3.7529 (12) Å between C1-ring and C1^i^-ring (symmetry code: (i) 1-x, 1-y, 1-z), and 3.6201 (12) Å between C11-ring and C11^ii^-ring (symmetry code: (ii) 2-x, 1-y, -z).

### Experimental

2, 2'-Methylenebis(4-chlorophenol) (1 g, 3.7 mmol) was dissolved in ethanol (30 ml). Potassium carbonate (1.5 g, 11 mmol) was added and the mixture was heated for an hour. Propargyl bromide (2 ml, 22 mmol) was added and the heating continues for another 3 h. Water (50 ml) was added. The organic compound was extracted by ethyl acetate (50 ml). Slow evaporation of ethyl acetate solution afforded crystals in 80% yield.

### Refinement

Carbon-bound H-atoms were placed in calculated positions [C—H 0.95 to 0.99 Å, *U*_iso_(H) 1.2*U*_eq_(C)] and were included in the refinement in the riding model approximation.

The acetylenic H-atoms were located in a difference Fourier map, and were refined with a distance of C–H 0.95±0.01 Å; their temperature factors were refined.

### Crystal data

**Table:**

C_19_H_14_Cl_2_O_2_	*Z* = 2
*M**_r_* = 345.20	*F*(000) = 356
Triclinic, *P*1	*D*_x_ = 1.436 Mg m^−^^3^
Hall symbol: -P 1	Mo *K*α radiation, λ = 0.71073 Å
*a* = 8.4844 (5) Å	Cell parameters from 3298 reflections
*b* = 9.7845 (6) Å	θ = 2.3–29.2°
*c* = 11.2568 (6) Å	µ = 0.41 mm^−^^1^
α = 86.258 (5)°	*T* = 100 K
β = 71.412 (5)°	Prism, colorless
γ = 64.707 (6)°	0.30 × 0.25 × 0.20 mm
*V* = 798.08 (8) Å^3^	

### Data collection

**Table:**

Agilent SuperNova Dual diffractometer with an Atlas detector	3523 independent reflections
Radiation source: SuperNova (Mo) X-ray Source	2975 reflections with *I* > 2σ(*I*)
Mirror	*R*_int_ = 0.029
Detector resolution: 10.4041 pixels mm^-1^	θ_max_ = 27.5°, θ_min_ = 2.3°
ω scans	*h* = −9→10
Absorption correction: multi-scan (*CrysAlis PRO*; Agilent, 2010)	*k* = −10→12
*T*_min_ = 0.815, *T*_max_ = 1.000	*l* = −11→14
6118 measured reflections	

### Refinement

**Table:**

Refinement on *F*^2^	Primary atom site location: structure-invariant direct methods
Least-squares matrix: full	Secondary atom site location: difference Fourier map
*R*[*F*^2^ > 2σ(*F*^2^)] = 0.038	Hydrogen site location: inferred from neighbouring sites
*wR*(*F*^2^) = 0.099	H atoms treated by a mixture of independent and constrained refinement
*S* = 1.00	*w* = 1/[σ^2^(*F*_o_^2^) + (0.0454*P*)^2^ + 0.2682*P*] where *P* = (*F*_o_^2^ + 2*F*_c_^2^)/3
3523 reflections	(Δ/σ)_max_ = 0.001
216 parameters	Δρ_max_ = 0.30 e Å^−^^3^
2 restraints	Δρ_min_ = −0.35 e Å^−^^3^

### Fractional atomic coordinates and isotropic or equivalent isotropic displacement parameters (Å^2^)

**Table:**

	*x*	*y*	*z*	*U*_iso_*/*U*_eq_	
Cl1	0.85099 (6)	0.23350 (5)	0.67998 (4)	0.02285 (13)	
Cl2	0.50877 (6)	0.78470 (5)	−0.01311 (4)	0.02450 (13)	
O1	0.55177 (16)	0.77464 (14)	0.41682 (11)	0.0196 (3)	
O2	1.00711 (16)	0.26968 (13)	0.18471 (11)	0.0182 (3)	
C1	0.3290 (3)	1.1163 (2)	0.32429 (18)	0.0228 (4)	
C2	0.3478 (2)	1.0165 (2)	0.39167 (16)	0.0182 (4)	
C3	0.3745 (2)	0.89566 (19)	0.47714 (16)	0.0175 (4)	
H3A	0.3701	0.9327	0.5584	0.021*	
H3B	0.2769	0.8603	0.4932	0.021*	
C4	0.6114 (2)	0.65014 (19)	0.48288 (15)	0.0158 (4)	
C5	0.5100 (2)	0.6355 (2)	0.60376 (16)	0.0176 (4)	
H5	0.3909	0.7132	0.6444	0.021*	
C6	0.5838 (2)	0.5070 (2)	0.66444 (16)	0.0176 (4)	
H6	0.5161	0.4960	0.7469	0.021*	
C7	0.7568 (2)	0.3954 (2)	0.60351 (16)	0.0176 (4)	
C8	0.8582 (2)	0.4087 (2)	0.48282 (16)	0.0172 (4)	
H8	0.9766	0.3300	0.4425	0.021*	
C9	0.7868 (2)	0.5366 (2)	0.42099 (15)	0.0154 (3)	
C10	0.8966 (2)	0.5563 (2)	0.29023 (15)	0.0161 (4)	
H10A	0.8890	0.6602	0.2891	0.019*	
H10B	1.0276	0.4843	0.2730	0.019*	
C11	0.8327 (2)	0.5312 (2)	0.18567 (15)	0.0151 (3)	
C12	0.7154 (2)	0.6527 (2)	0.13844 (16)	0.0170 (4)	
H12	0.6728	0.7530	0.1729	0.020*	
C13	0.6599 (2)	0.6288 (2)	0.04161 (16)	0.0172 (4)	
C14	0.7199 (2)	0.4852 (2)	−0.01130 (16)	0.0178 (4)	
H14	0.6819	0.4705	−0.0783	0.021*	
C15	0.8372 (2)	0.3614 (2)	0.03478 (16)	0.0167 (4)	
H15	0.8794	0.2615	−0.0005	0.020*	
C16	0.8922 (2)	0.38484 (19)	0.13286 (15)	0.0155 (3)	
C17	1.0784 (2)	0.1178 (2)	0.13135 (17)	0.0199 (4)	
H17A	1.1792	0.0504	0.1634	0.024*	
H17B	1.1309	0.1149	0.0389	0.024*	
C18	0.9368 (2)	0.0606 (2)	0.16123 (16)	0.0196 (4)	
C19	0.8221 (3)	0.0152 (2)	0.18736 (17)	0.0232 (4)	
H1	0.317 (3)	1.1950 (19)	0.2701 (17)	0.033 (6)*	
H19	0.729 (2)	−0.020 (3)	0.209 (2)	0.039 (6)*	

### Atomic displacement parameters (Å^2^)

**Table:**

	*U*^11^	*U*^22^	*U*^33^	*U*^12^	*U*^13^	*U*^23^
Cl1	0.0291 (3)	0.0206 (2)	0.0249 (2)	−0.0126 (2)	−0.01521 (19)	0.00937 (18)
Cl2	0.0240 (2)	0.0226 (3)	0.0224 (2)	−0.0051 (2)	−0.00986 (18)	0.00816 (18)
O1	0.0207 (6)	0.0165 (6)	0.0147 (6)	−0.0038 (5)	−0.0032 (5)	0.0024 (5)
O2	0.0191 (6)	0.0138 (6)	0.0207 (6)	−0.0033 (5)	−0.0100 (5)	−0.0003 (5)
C1	0.0210 (9)	0.0223 (10)	0.0237 (10)	−0.0072 (8)	−0.0089 (7)	0.0042 (8)
C2	0.0143 (8)	0.0201 (9)	0.0187 (9)	−0.0053 (7)	−0.0055 (7)	−0.0031 (7)
C3	0.0150 (8)	0.0176 (9)	0.0174 (9)	−0.0046 (7)	−0.0049 (7)	−0.0006 (7)
C4	0.0191 (9)	0.0154 (8)	0.0145 (8)	−0.0074 (7)	−0.0074 (7)	0.0012 (7)
C5	0.0173 (8)	0.0191 (9)	0.0161 (9)	−0.0080 (7)	−0.0046 (7)	−0.0002 (7)
C6	0.0200 (9)	0.0209 (9)	0.0151 (8)	−0.0120 (8)	−0.0056 (7)	0.0026 (7)
C7	0.0229 (9)	0.0185 (9)	0.0182 (9)	−0.0120 (8)	−0.0115 (7)	0.0052 (7)
C8	0.0166 (8)	0.0175 (9)	0.0194 (9)	−0.0075 (7)	−0.0077 (7)	0.0002 (7)
C9	0.0171 (8)	0.0182 (9)	0.0146 (8)	−0.0103 (7)	−0.0057 (6)	0.0004 (7)
C10	0.0156 (8)	0.0169 (9)	0.0161 (8)	−0.0074 (7)	−0.0049 (6)	0.0011 (7)
C11	0.0137 (8)	0.0191 (9)	0.0138 (8)	−0.0097 (7)	−0.0023 (6)	0.0019 (7)
C12	0.0177 (8)	0.0164 (9)	0.0153 (8)	−0.0091 (7)	−0.0011 (6)	0.0021 (7)
C13	0.0143 (8)	0.0197 (9)	0.0140 (8)	−0.0059 (7)	−0.0029 (6)	0.0061 (7)
C14	0.0155 (8)	0.0246 (10)	0.0141 (8)	−0.0093 (8)	−0.0050 (6)	0.0022 (7)
C15	0.0172 (8)	0.0177 (9)	0.0138 (8)	−0.0072 (7)	−0.0037 (6)	−0.0003 (7)
C16	0.0132 (8)	0.0180 (9)	0.0141 (8)	−0.0065 (7)	−0.0035 (6)	0.0035 (7)
C17	0.0188 (9)	0.0151 (9)	0.0213 (9)	−0.0023 (7)	−0.0068 (7)	−0.0026 (7)
C18	0.0232 (9)	0.0155 (9)	0.0151 (9)	−0.0029 (8)	−0.0070 (7)	−0.0012 (7)
C19	0.0279 (10)	0.0231 (10)	0.0172 (9)	−0.0107 (9)	−0.0056 (7)	−0.0002 (7)

### Geometric parameters (Å, °)

**Table:**

Cl1—C7	1.7518 (18)	C8—H8	0.9500
Cl2—C13	1.7492 (18)	C9—C10	1.521 (2)
O1—C4	1.374 (2)	C10—C11	1.516 (2)
O1—C3	1.435 (2)	C10—H10A	0.9900
O2—C16	1.378 (2)	C10—H10B	0.9900
O2—C17	1.432 (2)	C11—C12	1.389 (2)
C1—C2	1.183 (3)	C11—C16	1.400 (2)
C1—H1	0.94 (2)	C12—C13	1.385 (2)
C2—C3	1.462 (2)	C12—H12	0.9500
C3—H3A	0.9900	C13—C14	1.376 (3)
C3—H3B	0.9900	C14—C15	1.394 (2)
C4—C9	1.401 (2)	C14—H14	0.9500
C4—C5	1.395 (2)	C15—C16	1.394 (2)
C5—C6	1.389 (2)	C15—H15	0.9500
C5—H5	0.9500	C17—C18	1.470 (3)
C6—C7	1.379 (3)	C17—H17A	0.9900
C6—H6	0.9500	C17—H17B	0.9900
C7—C8	1.389 (2)	C18—C19	1.184 (3)
C8—C9	1.387 (2)	C19—H19	0.95 (2)
			
C4—O1—C3	117.28 (13)	C9—C10—H10A	108.7
C16—O2—C17	117.67 (13)	C11—C10—H10B	108.7
C2—C1—H1	178.8 (13)	C9—C10—H10B	108.7
C1—C2—C3	177.80 (19)	H10A—C10—H10B	107.6
O1—C3—C2	106.72 (13)	C12—C11—C16	118.08 (15)
O1—C3—H3A	110.4	C12—C11—C10	121.02 (15)
C2—C3—H3A	110.4	C16—C11—C10	120.89 (15)
O1—C3—H3B	110.4	C13—C12—C11	120.63 (16)
C2—C3—H3B	110.4	C13—C12—H12	119.7
H3A—C3—H3B	108.6	C11—C12—H12	119.7
O1—C4—C9	115.07 (14)	C14—C13—C12	121.33 (16)
O1—C4—C5	123.85 (15)	C14—C13—Cl2	119.55 (14)
C9—C4—C5	121.06 (16)	C12—C13—Cl2	119.12 (14)
C6—C5—C4	119.73 (16)	C13—C14—C15	119.11 (16)
C6—C5—H5	120.1	C13—C14—H14	120.4
C4—C5—H5	120.1	C15—C14—H14	120.4
C7—C6—C5	119.16 (16)	C14—C15—C16	119.73 (16)
C7—C6—H6	120.4	C14—C15—H15	120.1
C5—C6—H6	120.4	C16—C15—H15	120.1
C6—C7—C8	121.46 (16)	O2—C16—C15	123.84 (16)
C6—C7—Cl1	119.33 (13)	O2—C16—C11	115.05 (15)
C8—C7—Cl1	119.21 (14)	C15—C16—C11	121.11 (16)
C9—C8—C7	120.16 (16)	O2—C17—C18	112.48 (14)
C9—C8—H8	119.9	O2—C17—H17A	109.1
C7—C8—H8	119.9	C18—C17—H17A	109.1
C8—C9—C4	118.44 (15)	O2—C17—H17B	109.1
C8—C9—C10	121.38 (15)	C18—C17—H17B	109.1
C4—C9—C10	120.17 (15)	H17A—C17—H17B	107.8
C11—C10—C9	114.36 (13)	C19—C18—C17	178.90 (19)
C11—C10—H10A	108.7	C18—C19—H19	179.4 (15)
			
C4—O1—C3—C2	176.88 (13)	C9—C10—C11—C12	97.47 (18)
C3—O1—C4—C9	−178.72 (14)	C9—C10—C11—C16	−83.30 (19)
C3—O1—C4—C5	0.0 (2)	C16—C11—C12—C13	−0.2 (2)
O1—C4—C5—C6	−178.32 (15)	C10—C11—C12—C13	179.03 (15)
C9—C4—C5—C6	0.3 (3)	C11—C12—C13—C14	−0.5 (2)
C4—C5—C6—C7	−0.2 (3)	C11—C12—C13—Cl2	178.86 (12)
C5—C6—C7—C8	−0.2 (3)	C12—C13—C14—C15	0.8 (2)
C5—C6—C7—Cl1	179.66 (13)	Cl2—C13—C14—C15	−178.63 (13)
C6—C7—C8—C9	0.5 (3)	C13—C14—C15—C16	−0.2 (2)
Cl1—C7—C8—C9	−179.40 (12)	C17—O2—C16—C15	2.2 (2)
C7—C8—C9—C4	−0.3 (2)	C17—O2—C16—C11	−177.57 (14)
C7—C8—C9—C10	178.34 (15)	C14—C15—C16—O2	179.79 (15)
O1—C4—C9—C8	178.68 (14)	C14—C15—C16—C11	−0.5 (2)
C5—C4—C9—C8	−0.1 (2)	C12—C11—C16—O2	−179.54 (14)
O1—C4—C9—C10	0.0 (2)	C10—C11—C16—O2	1.2 (2)
C5—C4—C9—C10	−178.76 (15)	C12—C11—C16—C15	0.7 (2)
C8—C9—C10—C11	105.02 (18)	C10—C11—C16—C15	−178.53 (15)
C4—C9—C10—C11	−76.4 (2)	C16—O2—C17—C18	−71.59 (19)

### Hydrogen-bond geometry (Å, °)

**Table:**

Cg is the centroid of the C11–C16 benzene ring.

**Table:**

*D*—H···*A*	*D*—H	H···*A*	*D*···*A*	*D*—H···*A*
C6—H6···Cg^i^	0.95	2.60	3.471 (2)	153

Symmetry codes: (i) −*x*+1, −*y*+1, −*z*+1.
